# Opposing expression pattern of opsin 3 and opsin 5 in the developing and adult nasal epithelium

**DOI:** 10.1093/chemse/bjaf051

**Published:** 2025-11-07

**Authors:** Ramanujam Karthikeyan, Anna-Carin Hägglund, Ebba Bengtsson, Wayne I L Davies, Lena Gunhaga

**Affiliations:** Department of Medical and Translational Biology, Umeå University, Umeå, Sweden; Present address: Centre for Applied Research, Department of Biotechnology, Saveetha University, Tamil Nadu, India; Department of Medical and Translational Biology, Umeå University, Umeå, Sweden; Department of Medical and Translational Biology, Umeå University, Umeå, Sweden; Department of Medical and Translational Biology, Umeå University, Umeå, Sweden; Present address: Department of Molecular Biology, Umeå University, Umeå, Sweden; Department of Medical and Translational Biology, Umeå University, Umeå, Sweden

**Keywords:** opsin 3, opsin 5, olfactory, sensory epithelium, respiratory epithelium, mouse

## Abstract

In the nasal cavity, olfactory receptor neurons are situated in the sensory epithelium and act to transduce odor signals, whereas the respiratory epithelium is responsible for removing unwanted particles from inhaled air. Although several molecular markers have been identified to define multiple specific cell types in the sensory epithelium, less is known to indicate cells in the respiratory domain. We have recently shown that the non-visual photoreceptor opsin 3 (Opn3) is expressed in the developing olfactory region. This raised the question as to which functional role/s Opn3 might play in the nasal epithelium, as well as whether other non-visual photoreceptors may be expressed in this region. By using *Opn3*-*eGFP* and *Opn5*-tdTomato reporter mice in combination with Foxj1, Ker8, OMP, Sox2, and Tubb3 immunohistochemistry analyzes, our findings show that Opn3 is restricted to the olfactory sensory domain from early embryonic stages, whereas Opn5 is up-regulated in the respiratory epithelium at later developmental stages. In adulthood, Opn3 is expressed in Sox2/Ker8-positive sustentacular cells in the sensory epithelium, whereas Opn5 expression remains in the respiratory epithelium, thus indicating that these molecular markers could be used to distinguish the sensory versus respiratory epithelia. Studies of morphology and expression patterns of Foxj1, Ker8, OMP, Sox2, and Tubb3 in adult *Opn3*^−/−^ and *Opn5*^−/−^ mice did not reveal differences from wild-type mice. In addition, neither *Opn3*^−/−^ nor *Opn5*^−/−^ mice exhibited a disturbance in olfaction compared to wild-type littermates when performing a buried food test.

## 1. Introduction

The nasal epithelium is divided into sensory and respiratory epithelia, in which the sensory component consists of olfactory sensory neurons (OSNs) important for odor sensation, whereas the major function of the respiratory epithelium is to remove particles from inhaled air. The division into sensory and respiratory epithelia occurs already during early embryonic development (Maier et al. 2010). During early embryonic stages, several olfactory post-mitotic neurons leave the olfactory epithelium and migrate toward the forebrain (Fornaro et al. 2003; Maier et al. 2011), where some will form the terminal nerve ganglion and others will be included in the forebrain (Taroc et al. 2017; Palaniappan et al. 2019; Amato et al. 2024). During development, the sensory epithelium grows thicker due to neurogenesis and an increase in the number of cell layers, including horizontal basal and globose basal stem cells (HBCs and GBCs) in the basal surface of the epithelium, supporting sustentacular (Sus) cells in the apical surface, as well as immature and mature OSNs (Cau et al. 2002; Maier and Gunhaga 2009; Guo et al. 2010; Tucker et al. 2010; Krolewski et al. 2013). Also, in adulthood, neurogenesis is maintained in the sensory epithelium, where several studies have addressed neurogenic mechanisms (Maier et al. 2011; Kam et al. 2014; Panaliappan et al. 2018; McClintock et al. 2020), as well as odor receptor function (Dibattista et al. 2021; Hirota 2024). By contrast, the respiratory epithelium remains thin, with no or few neurons. To date, the molecular signature of the respiratory epithelium includes only a few identified markers (Maier et al. 2010; Amini et al. 2019; Larson et al. 2019).

Previous understanding of sensory receptors has expanded and it is now established that the expression pattern and function of sensory receptors are not solely confined to where they were initially identified, such as photoreceptors in the retina, olfactory receptors in the nasal apparatus, and taste receptors on the tongue (Dalesio et al. 2018). This is in agreement with findings that several olfactory receptors are expressed in the kidney and bladder, and taste receptors in the gastrointestinal and cardiovascular systems [reviewed in Papacocea et al. (2020), D'Urso and Drago (2021), Shepard (2021)]. Consistent with this, recent studies present a myriad of non-retinal structures that express one or more non-visual opsin (Opn) subclasses [reviewed in Moraes et al. (2021), Andrabi et al. (2023), Karthikeyan et al. (2023)]. The Opn family of photopigments are the predominant light-sensing molecules in many organisms, including humans, and for decades, it was assumed that ocular rods and cones of the retina were the only cells expressing light-sensitive photoreceptors in mammals. This view changed with the identification of Opn3, Opn4, and Opn5 photopigments in various cell types, where these opsins are predominantly not thought to be directly involved in image-forming vision, and therefore are known as non-visual opsins (Provencio et al. 1998; Blackshaw and Snyder 1999; Tarttelin et al. 2003). These opsin-based photopigments can respond to light: e.g. Opn3 has an absorption spectral maximum (i.e. λ_max_) at ∼465 nm (blue), and Opn5 at ∼380 nm in the ultraviolet A (UVA) range (Yamashita et al. 2010; Kojima et al. 2011; Kato et al. 2016; Sugihara et al. 2016). Functionally, previous studies have revealed diverse roles for Opn3 in the regulation of melanogenesis (Regazzetti et al. 2018; Ozdeslik et al. 2019), muscle relaxation (Yim et al. 2019; Wu et al. 2021), and thermogenesis (Nayak et al. 2020). In addition, Opn5 has been implicated in the vascular development of the eye (Nguyen et al. 2019), thermoregulation (Zhang et al. 2020), as well as circadian photoentrainment and the regulation of several circadian clock genes (Buhr et al. 2015; Buhr et al. 2019).

The present lack of reliable antibodies against murine Opn3 and Opn5 has led to the generation of distinct Opn3- and Opn5-reporter constructs to define the expression patterns of these opsins (Buhr et al. 2015; Olinski et al. 2020; Zhang et al. 2020; Davies et al. 2021). A recent study describing *Opn3*-eGFP expression in the developing head region of mice demonstrated that Opn3 is first expressed in the olfactory placode at embryonic day (E) 9.5, with an expanded *Opn3*-eGFP expression pattern that is maintained in the olfactory epithelium to at least E15.5 (Davies et al. 2021). However, the expression pattern of Opn3 in the nasal cavity during later embryonic/postnatal stages and during adulthood is currently unknown, as well as which specific cell type(s) express Opn3. Moreover, if other non-visual opsins are expressed in the nasal cavity and what potential function these opsins may exhibit in this region, have so far not been investigated. Here, we elucidate the expression patterns of Opn3 and Opn5 in the nasal cavity from embryonic to adult stages by using *Opn3*-*eGFP* and *Opn5*-tdTomato reporter mouse lines (Nguyen et al. 2019; Davies et al. 2021). Furthermore, the morphology of the nasal epithelium, the expression of several key molecular markers, as well as behavioral tests of olfaction, were analyzed in *Opn3*- and *Opn5*-deficient mice. Briefly, this study extends current knowledge of where and when key photoreceptors are expressed outside of the eye, thus implicating novel regions with the potential for photoreception, as well as defining a new marker for the respiratory olfactory epithelium.

## 2. Methods

### 2.1 Ethics declaration

Animal studies in the Gunhaga laboratory were approved by the Regional Animal Research Ethics Committee in Umeå, Sweden (Dnr A38-2019, A25-2024). All animal procedures were performed in accordance with the Umeå University animal care committee's regulations.

### 2.2 Animals and housing

Genetically modified mouse strains used were as follows: (i) an *Opn3*-*eGFP* reporter line (*Tg[Opn3-EGFP]JY3Gsat*; MMRRC/GENSAT stock number 030727-UCD, Mutant Mouse Resource & Research Centers, University of California, Davis, California); (ii) an *Opn5^Cre/+^*; *Ai14* reporter line (Nguyen et al. 2019); (iii) an *Opn3^lacZ/lacZ^* line that functionally acts like an *Opn3*^−/−^ line, mixed background strain C57Bl6/6N/FVB/N (Nayak et al. 2020); and (iv) an *Opn5*^−/−^ line, mixed background strain C57/129/CD1/FVB (Nguyen et al. 2019). Where relevant, *Opn3*-*eGFP*/*Opn5^Cre/+^*; *Ai14* double reporter mice were generated by crossing the *Opn3*-*eGFP* mice with the *Opn5^Cre/+^*; *Ai14* mouse line. Littermate mice were used as controls when studying *Opn3*^−/−^ and *Opn5*^−/−^ mice. All animals were housed at a 12 h:12 h light-dark (12L:12D) cycle in a temperature and humidity-controlled environment provided with a standard chow diet and water ad libitum.

*Opn3* ^+/+^ *, Opn3* ^−/−^, *Opn5*^+/+^, and *Opn5*^−/−^ mice used in behavior studies or sacrificed for tissue analyzes were housed in circadian cabinets (Actimetrics) in a reverse 12L:12D cycle regulated by Clocklab Chamber control software, in which light was switched on at 18:00 in the evening and switched off at 06:00 in the morning. Mice were kept in these cabinets for at least five weeks before behavioral studies or tissue analyzes were conducted. The circadian cabinets included white LEDs and additional specific LEDs of UVA (380 nm, 6.6 × 10^14^ photons cm^−2^ s^−1^), blue/green (453 and 523 nm, 3.0 × 10^15^ photons cm^−2^ s^−1^), and red (610 nm, 3.3 × 10^14^ photons cm^−2^ s^−1^), together giving a total photon flux of 8.9 × 10^15^ photons cm^−2^ s^−1^.

### 2.3 Genotyping

Primer sequences and oligonucleotide pairs for genotyping individual alleles for the different genetically modified mouse strains are listed in Supplementary Table S1, as previously described for *Opn3*-*eGFP* (Davies et al. 2021); *Opn3^lacZ/lacZ^* (*Opn3*^−/−^) (Nayak et al. 2020); *Opn5^Cre/+^*;*Ai14* and *Opn5*^−/−^ (Nguyen et al. 2019).

### 2.4 Tissue isolation and processing

Heads were isolated at Zeitgeber Time (ZT) 2–5 from three embryonic stages (E13.5, E15.5, and E17.5), as well as nasal cavity samples from postnatal stage (P) 14 and adults (3–6 months old). Embryonic tissue samples were immediately fixed in 4% paraformaldehyde (PFA) in phosphate buffer (PB) at 4 °C for 3 h to overnight (ON). Adult and P14 nasal tissues were isolated at ZT 2–5, dissected as described (Haglin et al. 2021), and fixed in 4% PFA at 4 °C for 2 to 5 h. In addition, adult tissue was thereafter decalcified in 0.5 M ethylenediamine tetraacetic acid (EDTA) at 4 °C for 3 days. Next, embryonic heads, postnatal and adult nasal tissues were cryo-protected in 30% sucrose, embedded in NEG-50 (Cellab, Stockholm, Sweden), and frozen. Samples were stored at −80 °C and cryo-sectioned with a thickness of 10 µm. At least 3 and up to 5 specimens at each time point were analyzed.

### 2.5 Immunohistochemistry

Immunohistochemistry was used by applying standard protocols (Wittmann et al. 2014) with the exception of Foxj1, Ker8, and Sox2 staining, which required antigen retrieval using 10 mM sodium citrate buffer (pH 9) at 85 °C for 25 min prior to antibody labeling. Primary antibodies used to detect specific markers were as follows: (i) mouse anti-Foxj1 (1:500; Thermo Fisher Scientific, Göteborg Sweden, #14-9965--82); (ii) chicken anti-GFP (1:600; Thermo Fisher Scientific, Göteborg, Sweden, #A600-101-215 M); (iii) rat anti-keratin 8 (TROMA-1; 1:50; Developmental Studies Hybridoma Bank); (iv) goat anti-OMP (1:1,000; FUJIFILM Wako Chemicals Europe GmbH, Neuss, Germany, #019-22291); (v) rabbit anti-Sox2 (1:1,000; Agrisera, Sweden); and (vi) rabbit anti-Tubb3 (1:1,000; BioLegend, San Diego, CA, USA, #802001). Secondary Alexa Fluor 488 antibodies (Thermo Fisher Scientific, Göteborg, Sweden) used were as follows: (i) a goat anti-chicken (1:600; #A-11039); (ii) a goat anti-rabbit (1:400; #A-11034); and (iii) a goat anti-mouse (1:400; #A-11001). Secondary Alexa Fluor 594 antibodies (Thermo Fisher Scientific, Göteborg, Sweden) used were: (i) a donkey anti-rabbit (1:400; #A-21207), (ii) a goat anti-mouse (1:400; #A-11032), and (iii) a donkey anti-goat (1:400; #A-11058). Also, a secondary Alexa Fluor 594 antibody from Jackson Immuno Research was used: a donkey anti-rat (1:400; 712-585-153). In all cases, 4′,6-diamidino-2-phenylindole (DAPI; 1:400; Sigma–Aldrich, Stockholm, Sweden) was included to counterstain nuclei. Slides were mounted with Aqua-Poly/Mount mounting medium (Polysciences, Warrington, PA, USA #18606) and stored at 4 °C.

### 2.6 *lacZ* staining

*lacZ* staining was essentially performed as previously described (Trifonov et al. 2016), using 1 mg/ml X-gal (5-Bromo-4-chloro-3-indolyl-β-galactopyranoside) and 0.4 mg/ml nitroblue tetrazolium (NBT) in the staining solution.

### 2.7 HCR Gold RNA-FISH in situ hybridization

Hybridization chain reaction (HCR) RNA fluorescence in situ hybridization (RNA-FISH) was performed using an *Opn5* probe and a fluorescently labeled amplifier (647 nm) (Molecular Instruments), following the HCR Gold RNA-FISH protocol from Molecular Instruments (https://files.molecularinstruments.com/HCR%E2%84%A2-Gold-RNAFISH-User-Guide.pdf).

### 2.8 Imaging

Immunohistochemically stained sections were examined by fluorescence microscopy (Nikon Eclipse, E800; Nikon Instruments Europe, Amsterdam, Netherlands), with images being captured using a Nikon DS-Ri1 digital camera and Nikon NIS-Elements F v4.6 software, an Axioscan Z1 slide scanner (Zeiss) or a SP8 FALCON confocal microscope (Leica), before being processed with Photoshop Creative Cloud (CC) 2025 (Adobe, San Jose, CA, USA).

### 2.9 Buried food test

The buried food test was essentially performed as previously described (Alberts and Galef 1971; Machado et al. 2018) at ZT 3–5. Prior to the buried food test, adult mice (3–6 months old), *Opn3*^+/+^ (*N* = 10), *Opn3*^−/−^ (*N* = 9), *Opn5*^+/+^ (*N* = 8) and *Opn5*^−/−^ (*N* = 11), of both sexes were habituated in the test cage for 30 min/day for five days. The day before the test, each mouse was food-deprived for ∼16 h, including the entire 12 h light phase, in clean cages provided with water and fresh bedding material. Three independent trials (T) were conducted with a minimum of 1 day up to 11 resting days with access to food and water, and always with the same number of resting days for individual groups of wild-types and *Opn3*- or *Opn5*-deficient mice tested. The buried food test was performed during the dark phase under dim red-light conditions in cages (42.5 cm × 27.6 cm × 15.3 cm) with 7 cm bedding material, where a piece of an Oreo cookie was hidden under the bedding material in the left corner (T1), middle (T2), or right corner (T3). Each mouse was tested separately and allowed to search for the cookie piece within a maximum test time of 10 min for each trial. Mice were video recorded (Sony FDR-AX53) during the trials and the time required to find the buried cookie piece was monitored.

### 2.10 Statistics

Statistical significances were calculated using unpaired two-tailed Student's *t*-tests, or a repeated measures two-way ANOVA followed by Sidak's multiple comparisons test.

## 3. Results

### 3.1 Restricted embryonic expression patterns of Opn3 and Opn5 in the nasal cavity

We have previously shown that Opn3 is up-regulated in the murine olfactory placode from E9.5, followed by expanded expression in the invaginating placode and developing olfactory epithelium up to E15.5 (Davies et al. 2021). To determine the expression pattern of Opn3 in more detail in the maturing olfactory epithelium, multiple stages of *Opn3*-*eGFP* embryonic, postnatal, and adult heads were collected and processed. Since Opn3 and Opn5 have been functionally linked with regard to their related, yet opposing downstream signaling pathways (Moraes et al. 2021; Andrabi et al. 2023; Karthikeyan et al. 2023), potential expression patterns of Opn5 in the nasal epithelium at different embryonic and adult stages were also examined. To detect the expression of Opn5, the reporter line *Opn5^Cre/+^*; *Ai14* was used, in which expression of tdTomato is driven by Cre-mediated recombination under the control of the *Opn5* promoter (Nguyen et al. 2019), herein referred to as *Opn5*-tdTomato. The nasal regions of the two reporter mouse lines were sectioned and used for immunohistochemistry. Herein, the *Opn3*-eGFP and *Opn5*-tdTomato expression will be referred to as Opn3 and Opn5, respectively. By E13.5, the invaginated olfactory epithelium has clearly become divided into distinctive sensory and respiratory regions (Maier et al. 2010). Moreover, within the olfactory epithelium, neurogenesis continues from earlier stages, where class III beta-tubulin (Tubb3) identifies post-mitotic immature OSNs and subsequently is a good marker to demarcate the sensory region of the olfactory epithelium (Fornaro et al. 2003; Maier et al. 2010; Coleman et al. 2019).

**Fig. 1.**
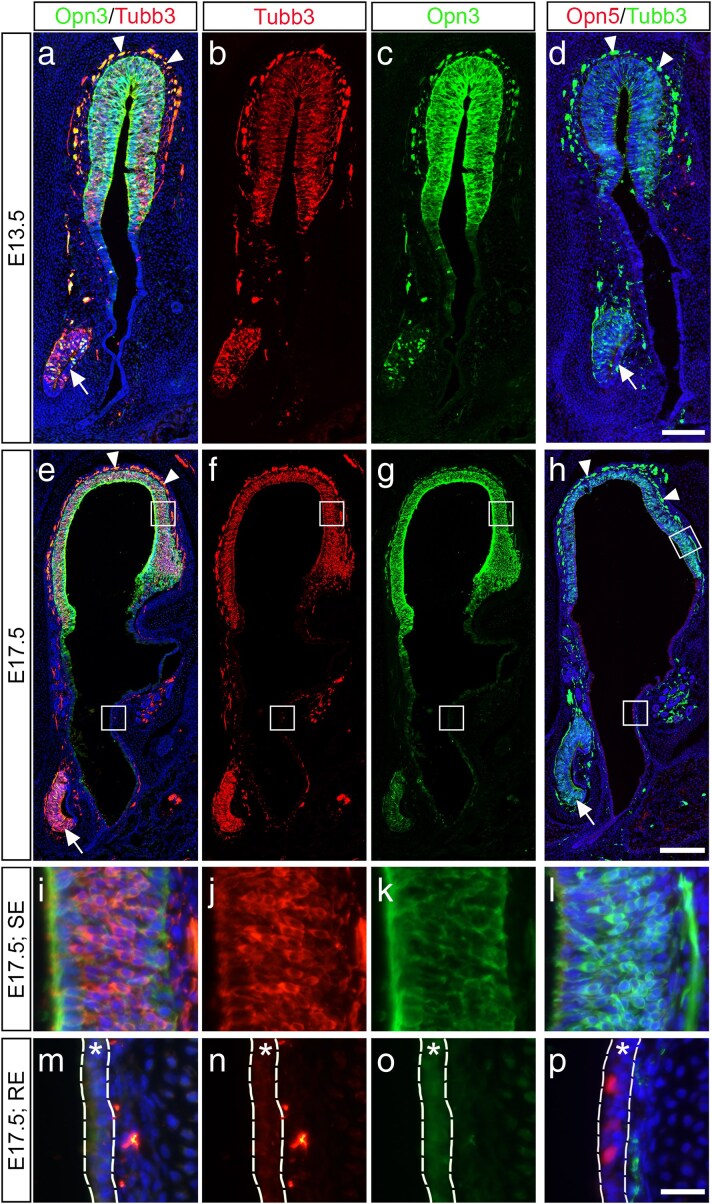
Immunohistochemistry analyzes of Opn3, Opn5, and Tubb3 expression in the nasal epithelium at E13.5 and E17.5 using *Opn3*-*eGFP* and *Opn5*-tdTomato mice. a–d) At E13.5, Opn3 is expressed in the Tubb3^+^ sensory epithelium (*N* = 3), while no Opn5 expression is detected in the entire nasal epithelium (*N* = 3). e–h) At E17.5, Opn3 is expressed in the Tubb3^+^ sensory epithelium (*N* = 5), and Opn5 is expressed in a few scattered cells in the respiratory epithelium (*N* = 5). White boxes indicate magnified images shown in (i–p). In (a, d, e and h), arrows indicate the vomeronasal organ, and arrowheads indicate migratory olfactory neurons. i–l) At e 17.5, Opn3 is co-localized with Tubb3, which marks postmitotic immature neurons, while no expression of Opn5 is detected in the sensory epithelium (SE). m–p) At E17.5, scattered Opn5 expression, but no Opn3 expression, is detected in the respiratory epithelium (RE; indicated with asterisks). Scale bars: a–d) 100 µm; e–h) 200 µm; i–p) 25 µm.

At E13.5, the sensory part of the epithelium, indicated by the post-mitotic neural marker Tubb3, has become thicker compared to the respiratory region, as indicated by DAPI staining (Fig. 1a, b, d). At this stage, Opn3 is co-localized with Tubb3 in the sensory part of the nasal epithelium, as well as in olfactory migratory neurons outside of the epithelium (Fig. 1a–c). Further, Opn3-reporter staining in the sensory epithelium was verified by *lacZ* staining in *Opn3*^lacZ/+^ olfactory epithelial sections (Supplementary Figure S1). By contrast, no Opn5-positive (Opn5^+^) cells were detected in the nasal epithelium at this stage (Fig. 1d).

To determine if and when the onset of Opn5 expression occurs in the nasal epithelium, later stages of embryonic nasal epithelia were examined. At E15.5, still no Opn5^+^ cells were detected in the nasal epithelium (Supplementary Figure S2). By E17.5, the entire nasal epithelium, including the respiratory region, has expanded. At this stage, a few scattered Opn5^+^ cells were observed in the respiratory domain (Fig. 1h), whereas Opn3 expression is maintained and located throughout the nasal sensory area (Fig. 1e–g). Further, Opn5-reporter staining in the respiratory epithelium was verified by HCR RNA-FISH staining using an *Opn5* mRNA probe (Supplementary Figure S3). Thus, during embryonic development, Opn3 is broadly expressed in the sensory region of the nasal epithelium, while limited Opn5 expression is restricted to the respiratory epithelium at E17.5.

A separate, yet relevant observation at E13.5 to 17.5 was that the vomeronasal organ, which is important for pheromone detection and located laterally to the respiratory epithelium (Mohrhardt et al. 2018; Murata et al. 2024), also expressed Opn3 and Tubb3, but not Opn5 (Fig. 1a–h).

### 3.2 Expanded Opn5 expression in the respiratory epithelium after birth

**Fig. 2.**
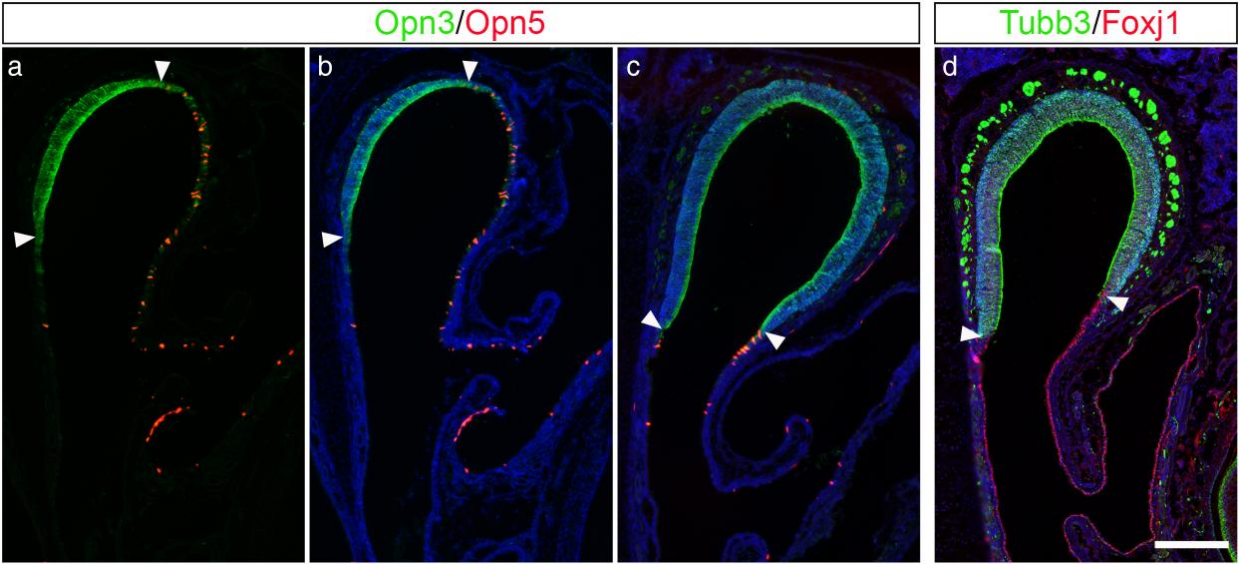
Analyzes of *Opn3*-eGFP/*Opn5*-tdTomato double reporter expression in comparison with Tubb3 and Foxj1 double immunohistochemistry at P14 (*N* = 4). a–c) At P14, the Opn5 expression has expanded in the respiratory epithelium, with opposing Opn3 expression observed in the sensory epithelium. Most Opn5^+^ respiratory epithelial cells were observed in the distal part of the nasal cavity. A few intermingled Opn3^+^ and Opn5^+^ cells were detected where the sensory and respiratory domains meet (indicated by arrowheads). d) The sensory epithelium defined by Tubb3 expression and the respiratory domain by Foxj1 expression mimic the Opn3 and Opn5 expression domains, respectively. Scale bar: 200 µm.

As mice are born more or less blind and with closed eyes, olfaction plays an essential role in recognizing the mother for receiving food and protection (Touj et al. 2020). Therefore, the expression patterns of Opn3 and Opn5 were examined shortly after birth, using P14 *Opn3*-*eGFP*/*Opn5*-tdTomato double reporter pups. At this stage, the nasal cavity has expanded further, with the expression pattern of Opn3 remaining restricted to the sensory epithelium as detected at embryonic stages (Fig. 2a–c). Moreover, at this stage, Opn5 expression has expanded into a slightly broader region of the respiratory epithelium, while still being detected in a scattered pattern, with the majority of the Opn5^+^ respiratory epithelial cells being observed in the distal part of the nasal cavity (Fig. 2a–c). Although the Opn3 and Opn5 expression patterns are opposing, a few intermingled Opn3^+^ and Opn5^+^ cells can be observed in the area where the sensory and respiratory epithelia meet (Fig. 2a–c). To verify this observation, P14 wild-type mice were used to analyze the expression of the general postmitotic immature neural marker Tubb3 together with Forkhead box protein J1 (Foxj1), the latter marker previously shown to be expressed in the adult respiratory epithelium (Larson et al. 2019). These results confirm a complementary expression pattern of Opn3 and Opn5 confined to the Tubb3^+^ olfactory sensory and Foxj1^+^ respiratory domain, respectively (Fig. 2c, d).

### 3.3 Opn3 and Opn5 expression in the nasal epithelium of adult mice

**Fig. 3.**
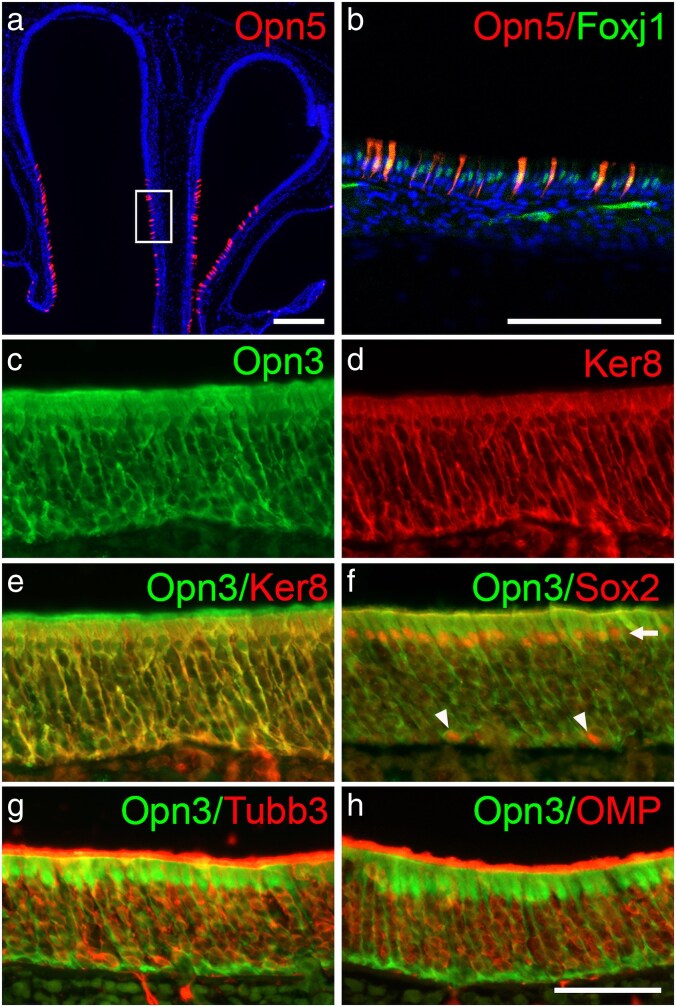
Immunohistochemistry analyzes of Opn3, Opn5, Foxj1, Ker8, Sox2, Tubb3, and OMP expression in the nasal epithelium at adulthood using *Opn3*-*eGFP* (*N* = 5) and *Opn5*-tdTomato mice (*N* = 4). a, b) In the adult nasal epithelium, an increased number of Opn5^+^ cells were detected in a scattered pattern within the Foxj1^+^ respiratory epithelium. White box in (a) indicates the magnified image shown in b. c–e) In the olfactory sensory epithelium, Opn3 and Ker8 are co-expressed in sustentacular cells. f) Opn3 is co-expressed with Sox2 in sustentacular cell nuclei (indicated by an arrow), but seemingly not in Sox2^+^ globose basal cells (indicated by arrowheads). g, h) Opn3 is not expressed in Tubb3^+^ postmitotic immature ORNs (g) or OMP^+^ mature ORNs (h). Scale bars: a) 200 µm; b) 100 µm; c–h) 50 µm.

In adults, an increase in the number of scattered Opn5^+^ cells in the Foxj1^+^ respiratory epithelium was detected (Fig. 3a, b). At this stage, a persistent broad expression pattern of Opn3 is observed in the neural sensory domain (Fig. 3c). Next, cell-specific expression of Opn3 in sustentacular cells were analyzed by the use of both keratin 8 (Ker8) and Sox2 (which belongs to the SoxB1 gene family) (Maurya et al. 2015; Packard et al. 2016), post-mitotic immature OSNs with Tubb3, mature OSNs by the use of olfactory marker protein [OMP (Keller and Margolis 1976)], and globose basal cells sustentacular cells by using Sox2 (Packard et al. 2016; Li et al. 2022). Imaging analyzes show that Opn3 expression is confined to Ker8^+^ and Sox2^+^ sustentacular cells with their cell bodies located at the apical side (Fig. 3c–f). Moreover, Sox2^+^ nuclei of globose basal cells at the basal side of the sensory epithelium are surrounded by GFP-positive structures (Fig. 3f), which could be sustentacular cells or the cytosol of globose basal cells. Since the cell bodies of globose basal cells lack clear GFP staining (Fig. 3f), Opn3 is seemingly not expressed in Sox2^+^ globose basal cells. Neither the Tubb3^+^ post-mitotic immature OSNs (Fig. 3g) nor the OMP^+^ olfactory sensory cells express Opn3 (Fig. 3h). Consistent with the known morphology of sustentacular cells (Maurya et al. 2015; McClintock et al. 2020), Opn3 and Ker8 cytoplasmic expression extend from the cell bodies at the apical side and through the epithelium, where the sustentacular cells closely surround and support immature and mature OSNs, as well as globose basal cells at the basal part of the olfactory epithelium (Fig. 3c–h).

### 3.4 Olfactory behavior tests in *Opn3*- and *Opn5*-deficient mice

**Fig. 4.**
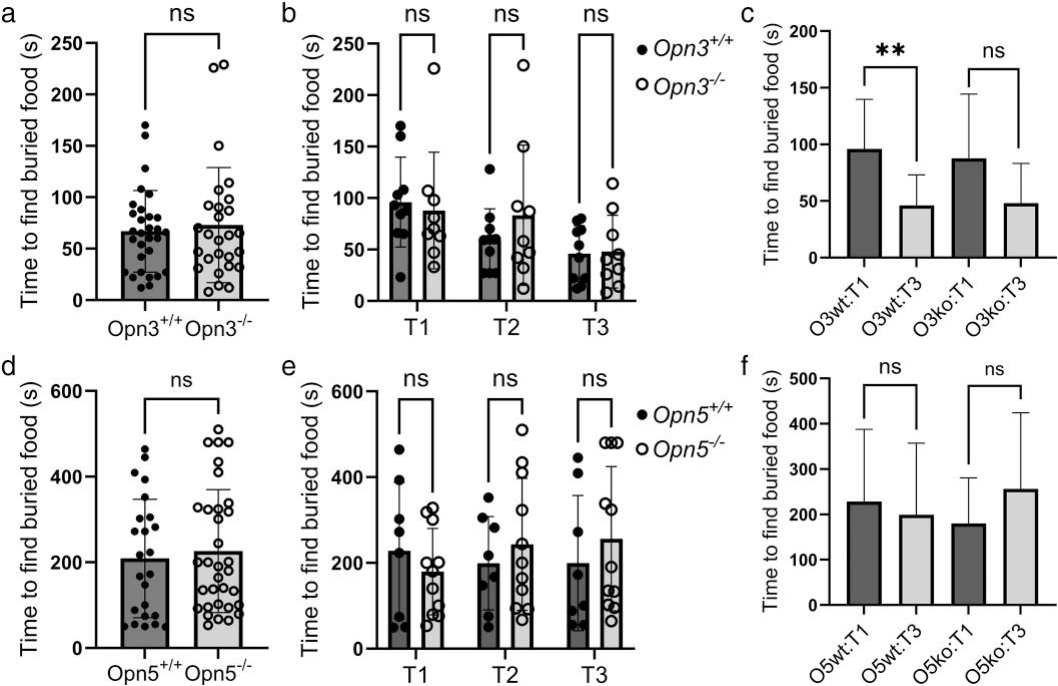
Buried food tests using *Opn3* or *Opn5* wild-type and deficient mice. Olfaction was evaluated by the buried food test, with a maximum of 10 min (i.e. 600 s) per trial to find a hidden cookie piece. a–c) The graphs show the time to find buried food for *Opn3*^+/+^ (*N* = 10) and *Opn3*^−/−^ mice (*N* = 9). a) The bars represent the average time of three independent trials. b) The bars represent the three individual trials, T1–T3, analyzed as a function of trial order. c) The bars represent time to find buried food between T1 and T3 for *Opn3*^+/+^ mice (*P* = 0.0076), and *Opn3*^−/−^ mice (*P* = 0.097). d–f) The graphs show the time to find buried food for *Opn5*^+/+^ (*N* = 8) and *Opn5*^−/−^ mice (*N* = 11). (d) The bars represent the average time of three independent trials. e) The bars represent the three individual trials, T1–T3. f) The bars represent time to find buried food between T1 and T3 for *Opn5*^+/+^ mice (*P* = 0.72) and *Opn5*^−/−^ mice (*P* = 0.21). Data on all graphs are presented as the mean ± standard deviation. Statistical significances were calculated using unpaired two-tailed Student's *t*-tests (a, c, d, f) or repeated measures two-way ANOVA followed by Sidak's multiple comparisons test (b, e).

The finding that Opn3 is broadly expressed in the adult sensory epithelium (Fig. 3c) suggests that Opn3 may play a functional role in odor sensation. A general key test to assess the ability of rodents to sense odors is the classic buried food test (Machado et al. 2018), first demonstrated in the early 1970s (Alberts and Galef 1971). Briefly, age-matched adult males and females, *Opn3*^+/+^ and *Opn3*^−/−^ mice, were food-deprived for ∼16 h, mostly during their inactive light phase. Thereafter, mice were olfactory challenged using a buried tasty cookie to find during their active dark phase, which was video recorded under infrared light conditions. Each mouse was placed in an individual clean test cage, followed by measurements of the time required for the mouse to actively find and grasp the cookie piece hidden beneath the 7 cm bedding material. The buried food tests were repeated three times, with a few days' rest in between, and in each of the three trials (T1–T3) the cookie pieces were hidden in a different place. Hiding the cookie piece in different locations was done to minimize the chance of a test mouse simply remembering where the buried food was located in previous tests. The results show no significant differences in the time taken to locate the buried cookie piece between *Opn3*^+/+^ and *Opn3*^−/−^ mice (Fig. 4a, b), suggesting that, in this test at least, olfaction is not affected by the lack of the Opn3 protein. Of interest, both *Opn3*^+/+^ and *Opn3*^−/−^ mice required less time in T3 compared to T1 to find the buried cookie (Fig. 4c), with a significant difference (*P* = 0.0076) for *Opn3*^+/+^ mice, but not for *Opn3*^−/−^ mice (*P* = 0.097). However, the nonsignificant results within the *Opn3*^−/−^ group compared to wild-type littermates most likely reflect a bigger variation in time between individual mice (Fig. 4c). This indicates a general functional memory process for this particular task, i.e. searching for a hidden piece of cookie, in the Opn3-strain, independent of *Opn3*-deficiency.

Although Opn5 expression is restricted to the respiratory epithelium, the buried food test was also used to examine general odor detection in *Opn5*^+/+^ and *Opn5*^−/−^ mice. Not surprisingly, the results did not reveal any time differences in locating the buried cookie piece between *Opn5*^+/+^ and *Opn5*^−/−^ mice (Fig. 4d–f). Notably, the Opn5-strain did not appear to demonstrate a learning or memory process between T1 and T3 to find the cookie pieces (Fig. 4f; *P* = 0.5). This apparent lack of learning or memory processes in *Opn5* mice, as well as the marked disparities in time to find buried food between *Opn3* and *Opn5* mice, are most likely due to strain differences. Since strain differences are known to have an impact on behavioral studies (Voikar et al. 2001), this prohibits direct comparisons between these two strains. Taken together, the use of the buried food test did not reveal any obvious perturbation of olfaction in either *Opn3* or *Opn5-*deficient mice compared to wild-type littermates.

### 3.5 Analyzes of the nasal cavities in Opn3- and Opn5-deficient mice

So far, results presented show clear distinctions in Opn3 and Opn5 expression in the sensory versus respiratory nasal epithelia, respectively, but neither *Opn3-* nor *Opn5*-deficient mice exhibited any defects in finding hidden food. Therefore, the expression patterns of a set of physiologically relevant molecular markers (Tubb3, Foxj1, OMP, Sox2, and Ker8) were analyzed in wild-type controls and *Opn3* and *Opn5*-deficient adult mice (Nguyen et al. 2019; Nayak et al. 2020).

**Fig. 5.**
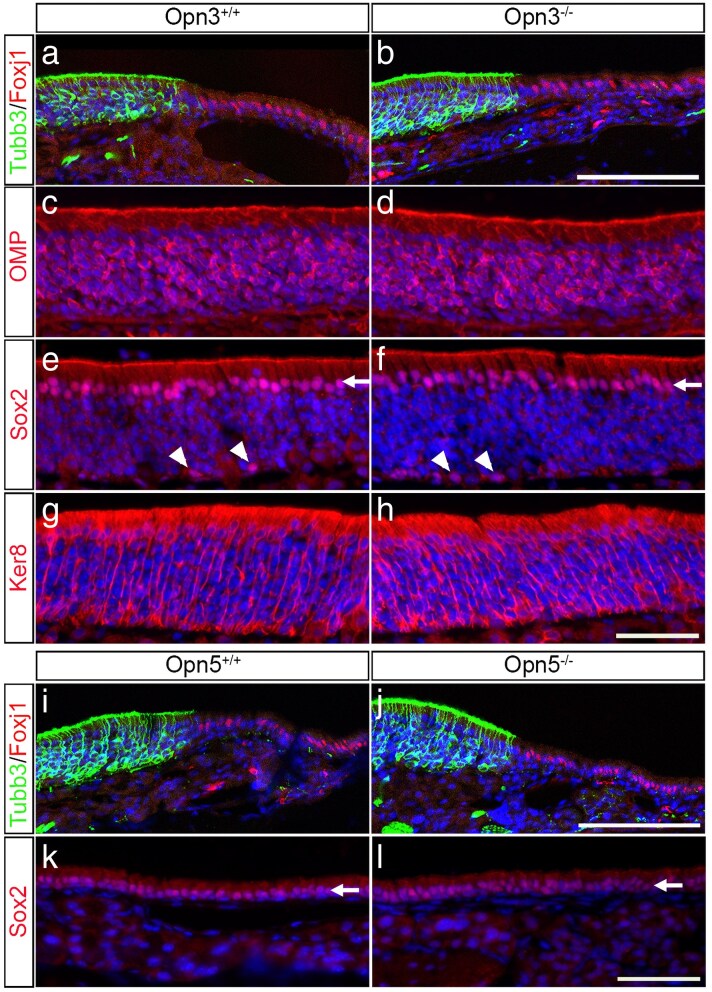
Immunohistochemistry analyzes of Tubb3, Foxj1, OMP, Sox2 and Ker8 in the nasal cavity of adult *Opn3*^+/+^, *Opn3*^−/−^, *Opn5*^+/+^, and *Opn5*^−/−^ mice (*N* = 3 for each mouse genotype). a–h) No apparent differences in the expression of Tubb3, Foxj1, OMP, Sox2, or Ker8 in the sensory epithelium of *Opn3*^−/−^ compared to *Opn3*^+/+^ mice. e, f) Arrows indicate the Sox2^+^ sustentacular cells, and arrowheads indicate Sox2^+^ globose basal cells. i–l) No apparent difference in the expression of Tubb3, Foxj1, or Sox2 in the respiratory epithelium of *Opn5*^−/−^ compared to *Opn5*^+/+^ mice. k, l) Arrows indicate the Sox2^+^ positive cells in the respiratory cell layer. Scale bars: a, b, i, j) 100 µm; c–h, k, l) 50 µm.

In adult mice, analyzes of DAPI and hematoxylin–eosin staining indicated no apparent differences in either morphology or histology of the nasal cavities of *Opn3*^+/+^ and *Opn3*^−/−^, as well as *Opn5*^+/+^ and *Opn5*^−/−^ mice, respectively (Fig. 5 and Supplementary Figures S4, S5). Further, the expression patterns of Tubb3, which defines postmitotic immature neurons in the sensory epithelium, and Foxj1, which marks the respiratory region, were similar between wild-types and *Opn3* and *Opn5*-deficient mice (Fig. 5a, b, i, j). This suggests that neither Opn3 nor Opn5 is required for the general division of the nasal epithelium into a sensory region and a respiratory zone. The expression of OMP^+^ OSNs in the nasal cavity was also similar between wild-types and *Opn3*-deficient mice (Fig. 5c, d), as well as the expression patterns of Sox2^+^ and Ker8^+^ sustentacular cells and Sox2^+^ globose cells (Fig. 5e–h). Moreover, wild-types and *Opn5*-deficient mice exhibited a similar Sox2 expression pattern in the respiratory epithelium (Fig. 5k, l). Together, these data indicate that although Opn3 and Opn5 have distinct expression patterns in the sensory and respiratory epithelia, genetic deletion of either of the opsin genes does not alter the overall morphology of the nasal epithelium or the expression patterns of Sox2, Ker8, Tubb3, OMP, or Foxj1 in the adult nasal cavity.

## 4. Discussion

Several markers are known to define the neural sensory olfactory epithelium, including the various cell types within [reviewed in Murdoch and Roskams (2007), McClintock et al. (2020)], while only a few molecular markers have been identified to be restricted to the respiratory domain (Maier et al. 2010; Amini et al. 2019; Larson et al. 2019). Now, this study identifies Opn5 as a potential new marker of the respiratory nasal epithelium, with an opposing expression pattern to Opn3, which is expressed in the sensory olfactory epithelium. Other studies have also reported disparate expression patterns of these opsins, as well as opposing roles for Opn3 and Opn5 in other systems. For example, in adipose tissues, Opn3 is expressed in white adipocytes where it is required to enhance thermogenesis (Nayak et al. 2020). By contrast, Opn5-positive neurons located in the hypothalamic preoptic area have been shown to regulate thermogenesis by decreasing the activity in brown adipose tissue (Zhang et al. 2020). Consistently, *Opn3*-deficient mice exhibited diminished thermogenesis during cold exposure, whereas mice lacking *Opn5* showed exaggerated thermogenesis (Nayak et al. 2020; Zhang et al. 2020). Moreover, a different physiological example shows that *Opn3* decreases melanin production (Dong et al. 2022), while *Opn5* is implied in the induction of melanocyte development and melanin synthesis (Lan et al. 2021).

Findings here show a widespread expression pattern of Opn3 in the sensory epithelium at key embryonic and postnatal stages. At adult stages, Opn3 is co-expressed with Ker8 and Sox2 in sustentacular cells surrounding the OSNs, but not in Sox2-positive globose basal cells. The cytoplasmic GFP protein under the regulation of the *Opn3* promoter allowed for detailed visualization of the sustentacular cells, showing that the Opn3^+^ cells were intermingled with and enwrapped OSNs. Consistently, physiological studies, including intracellular Ca^2+^ recordings, have implicated that interactions between sustentacular and olfactory sensory cells can occur via gap junctions (Vogalis et al. 2005; Hegg et al. 2009). Moreover, several studies have supported a role for sustentacular cells and gap junctions in modulating the olfactory sensitivity of OSNs (Hegg et al. 2009; Zhang 2010; Le Bourhis et al. 2014). It is also known that sustentacular cells contribute to generating specific mucus that surrounds the OSNs, which support odor transduction (Villar et al. 2017; Acevedo et al. 2019). In our study, analyzes of Opn3-deficient mice did not indicate a loss or apparent alteration of Sox2 or Ker8 expression in sustentacular cells.

OMP has previously been shown to be required for proper odor quality sensitivity using an odorant confusion matrix task in OMP knock-out and control mice (Youngentob et al. 2001). Moreover, in two mouse models of allergic rhinitis, OMP expression in the olfactory epithelium is reduced and accompanied by an increased time period to find hidden pellets in the buried food test, compared to control mice (Wang et al. 2017; Jung and Kim 2020). In another mouse disease model [i.e. the experimental autoimmune encephalomyelitis (EAE)], the expression of OMP was significantly lowered in the olfactory epithelium, accompanied by significantly more time required by the EAE mice to find pellets in the buried food test, compared with the same mice one month before neuroinflammatory onset (Kim et al. 2018). Collectively, these results suggest that reduced olfaction measured by increased time to find buried food can be linked to a downregulation of OMP. In the present study, the behavioral response in odor sensation, in terms of the ability to find buried food, does not appear to be dependent on Opn3 function. This result is supported by the seemingly unaltered morphology of the olfactory sensory epithelium, including a similar expression pattern of both Sox2, Ker8, and OMP in *Opn3*-deficient mice, compared to wild-type littermates. Finding food through odor sensation and eating habits are connected, and in both mice and humans, it has been shown that olfaction is, in part, regulated by hunger, which in turn affects feeding behavior (Nie et al. 2023; Stark et al. 2024). Previous studies have observed that *Opn3*-deficient mice tend to eat less than wild-types, which suggests that Opn3 activity might promote food consumption (Nayak et al. 2020; Haddad et al. 2025). Now our findings indicate that this reduction in food consumption in *Opn3*-deficient mice does not appear to be due to disturbed olfaction, at least not when tested by the buried food test.

Future studies will show whether Opn3-dependent feeding behaviors are entirely independent of olfaction or whether Opn3 may have indirect functional roles if mice are challenged in other olfaction-related behavioral tests. It is possible that key opsins in the nasal epithelium, such as Opn3 and Opn5, may play a role in the photoentrainment of certain aspects of the olfactory sensory system or respiratory system, respectively, which remains to be evaluated. Another possibility is that Opn3 and/or Opn5 may influence other, yet untested, physiological systems and behaviors such as pheromone-dependent mate selection, parental-pup bonding/rearing interactions or prey–predator relationships, some of which may or may not be light-dependent.

## 5. Conclusions

Results from this study clearly show broad expression of Opn3 in the developing and adult olfactory sensory epithelium, and specifically, Opn3 is co-expressed with Ker8 and Sox2 in sustentacular cells at adult stages. By contrast, a scattered expression pattern was observed for Opn5 in the Foxj1^+^ respiratory nasal epithelium, which also expressed Sox2. No apparent differences in olfaction were observed in either *Opn3* or *Opn5*-deficient mice using the buried food test. Thus, whether photo-activation of Opn3 and/or Opn5 affects olfaction in a more subtle manner or mucus production, respectively, remains to be understood. Another possibility is that Opn3 and/or Opn5 may provide circadian information about light versus dark phases to the olfactory bulb. In support of this hypothesis, it has been shown that the olfactory bulb exhibits a circadian rhythm linked to sniff cycles, which in turn has been linked to memory formation (Heck et al. 2019). Taken together, continued studies are required to determine the elusive function of the non-visual opsins Opn3 and Opn5 expressed in the nasal epithelium.

## Supplementary Material

bjaf051_Supplementary_Data

## Data Availability

Data will be made available upon request from the corresponding author.
