# Supplementary material for: Opposing expression pattern of opsin 3 and opsin 5 in the developing and adult nasal epithelium
Source: Chem Senses. 2025 Nov 7;50:bjaf051. doi: 10.1093/chemse/bjaf051 (PMC12628503; doi:10.1093/chemse/bjaf051)
Supplement: bjaf051_Supplementary_Data [file bjaf051_supplementary_data.docx]

**SUPPLEMENTARY MATERIAL**

**FIGURES**

**
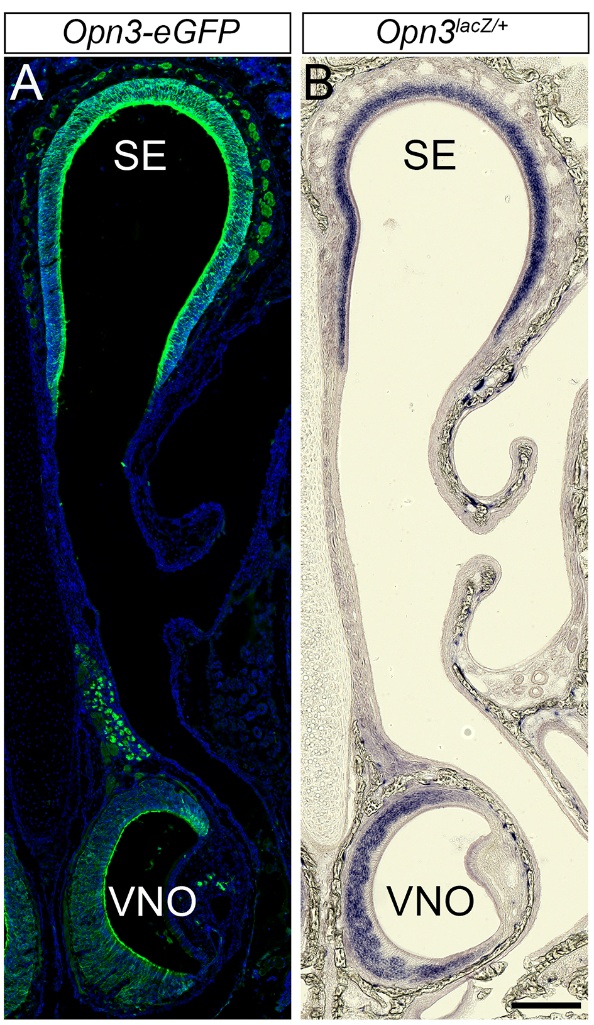
**

**Suppl. Fig. 1.** Validation of *Opn3*-eGFP reporter expression in the sensory epithelium. A,B) At P14, *lacZ* staining in *Opn3*^lacZ/^*^+^* nasal epithelial sections (B) mimicked *Opn3*-eGFP expression in the olfactory sensory epithelium (SE), as well as in the vomeronasal organ (VNO) (A). Scale bar: 200 µm.

**
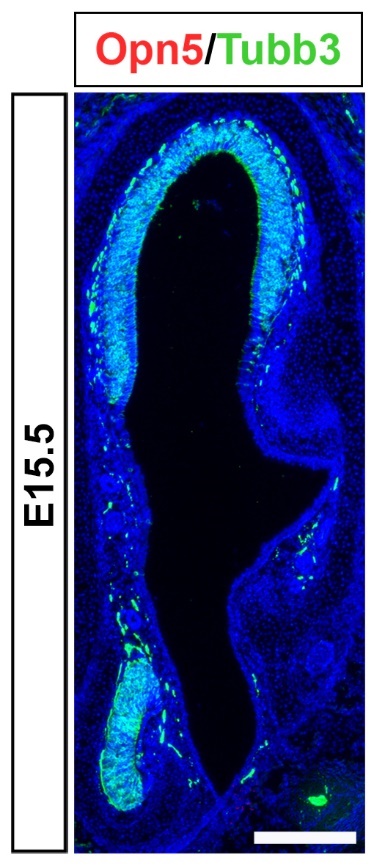
**

**Suppl. Fig. 2.** No Opn5 expression in the nasal epithelium at E15.5. Immunohistochemistry analyzes of Opn5 and Tubb3 expression in the nasal epithelium at E15.5, using *Opn5*-tdTomato mice, indicate the absence of Opn5^+^ cells in the nasal epithelium, while Tubb3 is expressed broadly in the sensory epithelium (N = 5). Scale bar: 200 µm.


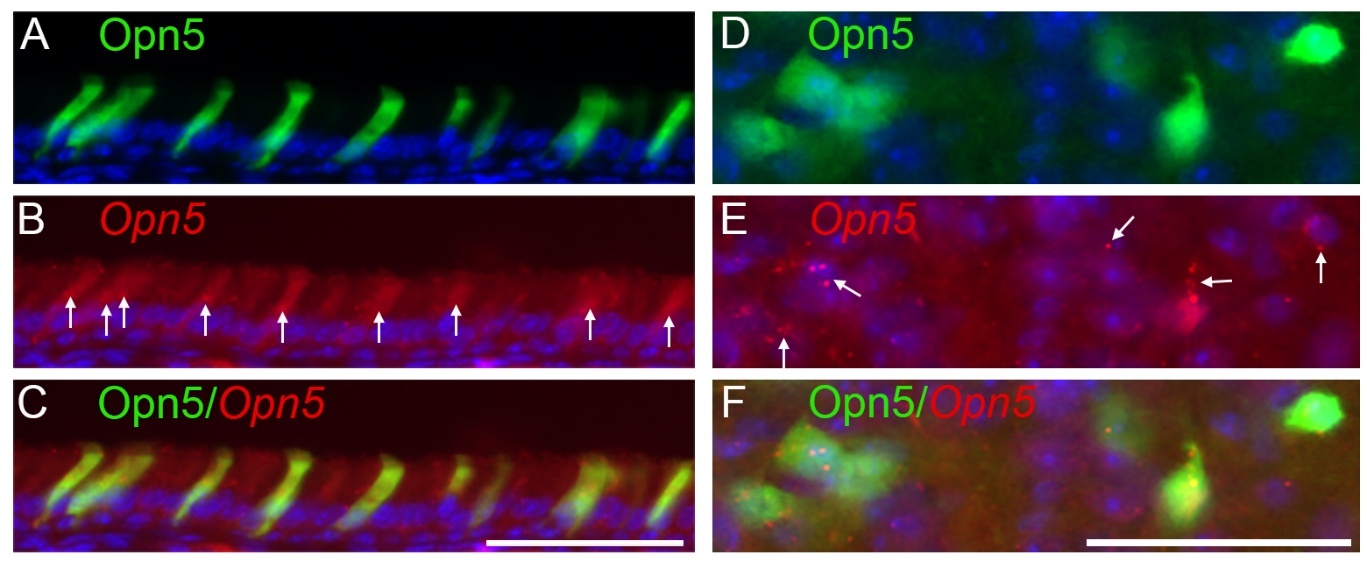


**Suppl. Fig. 3.** Validation of *Opn5*-tdTomato reporter expression in the respiratory epithelium. A-F) *Opn5* mRNA staining detected by HCR RNA-FISH (pseudo-colored in red and indicated by arrows in B and E) overlaps with *Opn5*- tdTomato reporter expression (pseudo-colored in green). A-C) At P14, *Opn5* mRNA staining overlaps with *Opn5*- tdTomato reporter expression in the olfactory respiratory epithelium. D-F) HCR RNA-FISH *Opn5* mRNA staining is verified by overlapping labeling with *Opn5*- tdTomato reporter expression in the preoptic area in the hypothalamus of adult mice (Zhang et al., 2020; D´Souza et al., 2021). Scale bars: 50 µm.

**
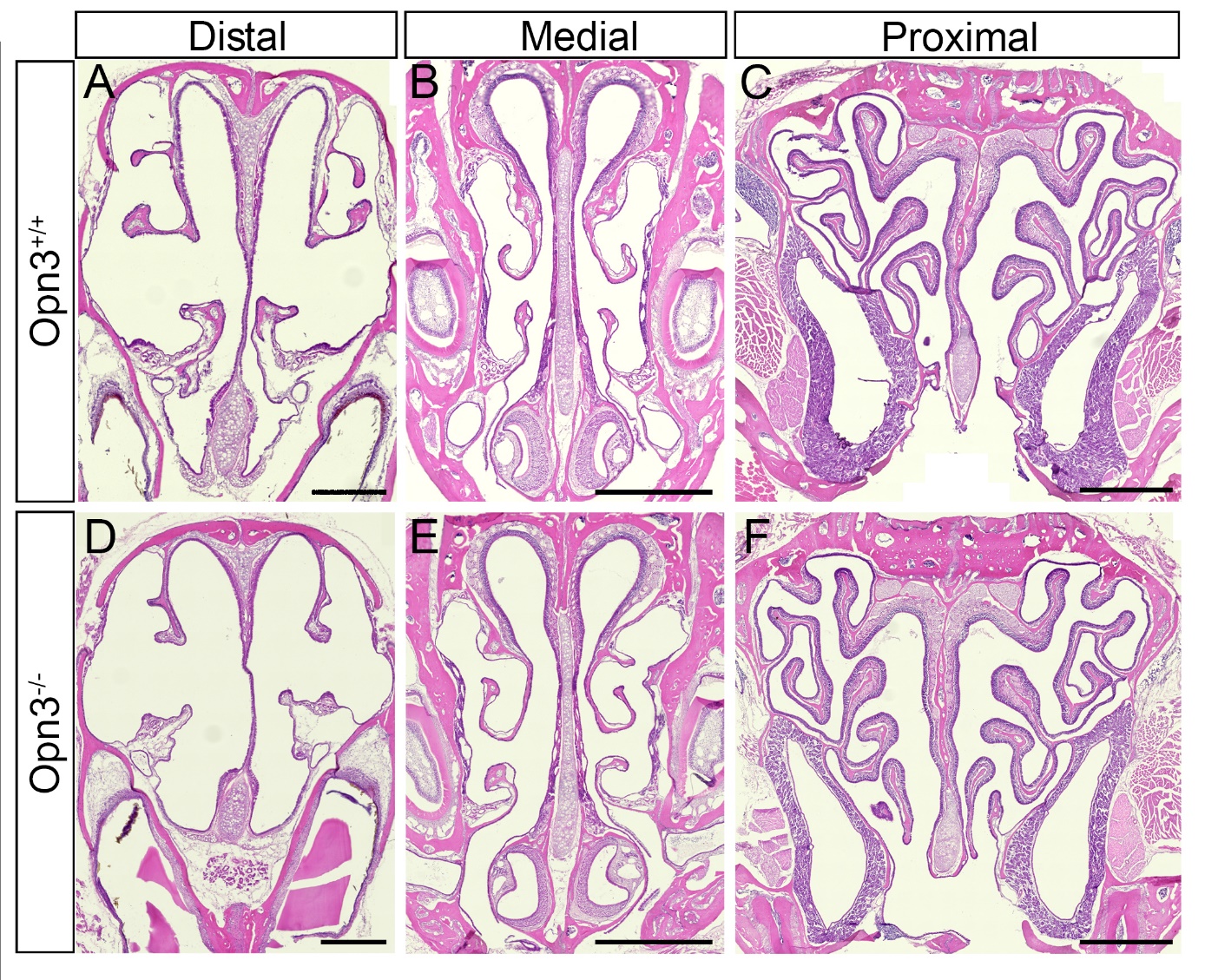
Suppl. Fig. 4.** A-F) Hematoxylin-eosin staining did not indicate apparent differences in the morphology of the nasal epithelium of adult *Opn3*^-/-^ mice throughout the nasal cavity (D-F) compared to wild type-mice (A-C). Scale bars: A,D 500 µm; B,C,E,F 1000 µm.

**
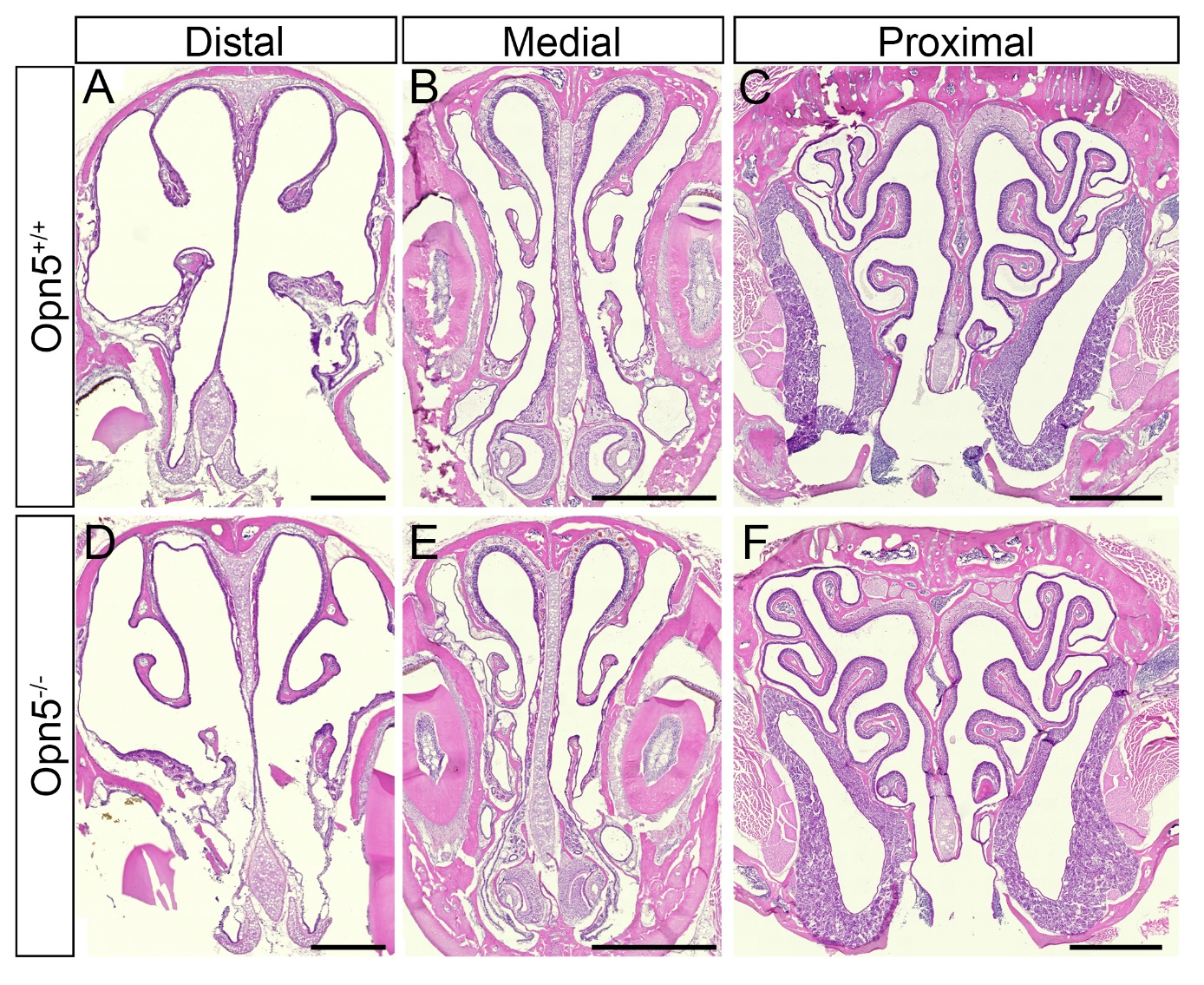
Suppl. Fig. 5.** A-F) Hematoxylin-eosin staining did not indicate apparent differences in morphology of the nasal epithelium of adult *Opn5*^-/-^ mice throughout the nasal cavity (D-F) compared to wild-type mice (A-C). Scale bars: A,D 500 µm; B,C,E,F 1000 µm.

**TABLE**

**Supplementary Table 1.** Genotyping primer sequences and pairs for alleles to verify specific mouse genotypes.

| Allele | Forward (F) and Reverse (R) Primers (5' to 3') | Primer Pairs | bp |
| --- | --- | --- | --- |
| Opn3 | F1: ACCCAGGCTTCTTTTGGTCT  R1: AGAGTCGTTGGCATCCTTGG  F2: ACTATCCCGACCGCCTTACT  R2: GAACTGATGGCGAGCTCAGA | F1R1 – Wildtype  F2R2 – Opn3^-^ | 1191  701 |
| Opn5 | F1: CACAGTATGTGTGACAACCT  R1: GTGGACAGATTAACTGAAGC  R2: GAACTGATGGCGAGCTCAGA | F1R1 – Wildtype  F1R2 – Opn5^-^ | 546  376 |
| Opn3^egfp^ | F1: ACCCAGGCTTCTTTTGGTCT  R1: AGAGTCGTTGGCATCCTTGG  F2: CAGAGCGTGAGATCCACCCTGTT  R2: TAGCGGCTGAAGCACTGCA | F1R1 – Wildtype  F2R2 – Opn3^egfp^ | 1191  320 |
| Opn5^cre^ | F1: TGGAAAGAGATGCATTTGTGAG  F2: CACTGCATTCTAGTTGTGGTTTGTCC R1: ACAGCCTATGAATTCTCTCAATGC | F1R1 – Wildtype  F2R1 – Opn5^cre^ | 300  209 |

**References**

Zhang, K.X., S. D'Souza, B.A. Upton, S. Kernodle, S. Vemaraju, G. Nayak, K.D. Gaitonde, A.L. Holt, C.D. Linne, A.N. Smith, N.T. Petts, M. Batie, R. Mukherjee, D. Tiwari, E.D. Buhr, R.N. Van Gelder, C. Gross, A. Sweeney, J. Sanchez-Gurmaches, R.J. Seeley, and R.A. Lang. 2020. Violet-light suppression of thermogenesis by opsin 5 hypothalamic neurons. *Nature*. 585:420-425.

D’Souza, S.P., D.I. Swygart, S.R. Wienbar, B.A. Upton, K.X. Zhang, R.D. Mackin, A.K. Casasent, M.A. Samuel, G.W. Schwartz, and R.A. Lang. 2021. Retinal patterns and the cellular repertoire of neuropsin (Opn5) retinal ganglion cells. J Comp Neurol. 530:1247–1262.
